# Dental Trauma Management in a Young Teenager through Endodontics and Implantology: A Case Report

**DOI:** 10.3390/healthcare9050542

**Published:** 2021-05-06

**Authors:** Marie-Laure Munoz-Sanchez, Nicolas Decerle, Laurent Devoize, Emmanuel Nicolas, Pierre-Yves Cousson, Jean-Luc Veyrune

**Affiliations:** 1 Faculty of Dentistry, University of Clermont Auvergne, 63000 Clermont-Ferrand, France; nicolas.decerle@uca.fr (N.D.); laurent.devoize@uca.fr (L.D.); emmanuel.nicolas@uca.fr (E.N.); p-yves.cousson@uca.fr (P.-Y.C.); j-luc.veyrune@uca.fr (J.-L.V.); 2 CHU Clermont-Ferrand, Service d’Odontologie, 63003 Clermont-Ferrand, France

**Keywords:** dental traumatology, adolescence, endodontics, mini-implant

## Abstract

Endodontic treatment is often the first-line procedure to manage the immediate or long-term aftermath of dental trauma, particularly in cases of luxation or avulsion. Failure to manage trauma in the short or medium term leads to significant functional or aesthetic consequences, especially in the adolescence period. Under this specific conditions, endodontic treatment could provide a temporary solution by keeping teeth with poor prognosis on the arch while waiting for better anatomical conditions for implantology. This clinical case aimed to describe the management of a maxilla-facial dental trauma and the following consequences in a 10-year-old male patient. Clinical and radiological examination showed complete extrusive luxation of 11 and 21 and intrusive luxation of 12 and 22. Endodontic treatment of 11 and 21 was performed six months after the trauma. Two years later, the patient was referred to the endodontic department because pink spot lesions appeared on 12 and 22 due to cervical invasive resorptions (class III for 12 and class II for 22). Endodontic treatment of 12 and filling with resin composite of 22 were performed. During the following two years, complication management finally led to placement of four OBI^®^ (Euroteknika, Sallanches, France)-type mini-implants after avulsion of all four maxillary incisors. Palliative endodontic treatment helped maintain the prosthetic space and the volume of supporting tissue needed for future implant placement. The interest of using delaying procedures (palliative endodontic treatments and mini-implants) was to allow the patient to complete growth. Managing early treatment failure of trauma in adolescents has to be pluridisciplinary and should take into account the evaluation of the treatment’s difficulty, the prognosis of the endodontic treatment, the available bone volume and the pubertal growth stage.

## 1. Introduction

Dento-alveolar trauma is the most common type of facial trauma [1]. The maxilla is the region most affected by trauma during childhood or adolescence [2], with 75% of facial trauma occurring during this period [3]. The teeth most affected are the maxillary central incisors, followed by the maxillary lateral incisors and the mandibular incisors [4]. The prognosis for traumatized teeth depends mainly on the type of trauma: the survival rate for reimplanted permanent teeth is 50% at 5.5 years [5], while the risk of pulpal necrosis is 25% for teeth with a coronal fracture associated with dislocation [6]. The prognosis of traumatized teeth also depends on the development stage of root construction: reimplanted immature teeth have a lower survival rate than reimplanted mature teeth [7]. In traumatology, the initial dental or pulpal diagnosis, the strict application of treatment protocols and the importance of follow-up are key elements of success. Endodontic treatment is often the first-line procedure to manage the immediate or long-term aftermath of dental trauma, particularly in cases of luxation or avulsion [8,9]. In the event of failure to manage the trauma in the short or medium term, the functional or aesthetic consequences can be significant, especially in the adolescence period. The implant is the solution of choice to compensate for edentulism in adults [10,11]; however, it is more controversial in adolescents, even if the use of implantology is increasingly recommended in very specific cases, traumatic or otherwise [12,13]. Thus, managing complications or failure will depend on the age of the patient, the type of teeth involved (deciduous or permanent teeth) and the extent of the lesions [14]. The individual dental prognosis and the benefit/risk ratio will have to be considered within a multidisciplinary team in order to guide decisions on the avulsion or conservation of traumatized teeth in a child or young adolescent. Indeed, it may be important to keep the traumatized teeth in order to preserve the bone capital for rehabilitation with implants. Endodontic treatment could thus provide a temporary solution that, by keeping teeth on the arch, will help maintain the prosthetic space and the volume of supporting tissue necessary for future implant placement. This clinical case aimed at describing the management of a maxilla-facial dental trauma in a 10-year-old male patient, and its following consequences over the course of 5 years.

## 2. Case Report

### 2.1. Traumatology and Treatment

**Figure 1:**
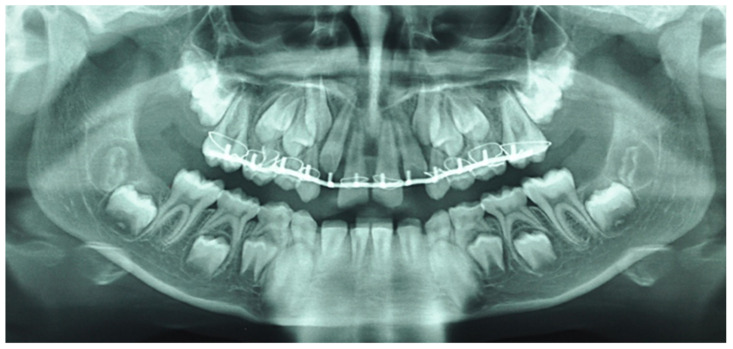
Orthopantomogram of the patient’s initial dental condition in October 2015.

**Figure 2:**
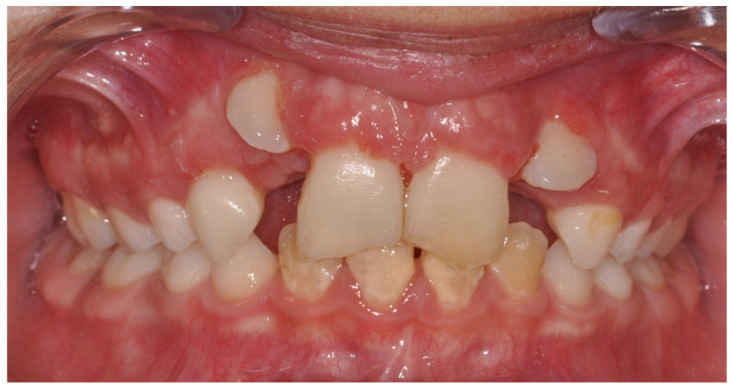
Endo-buccal view of the initial situation in October 2015.

**Figure 3:**
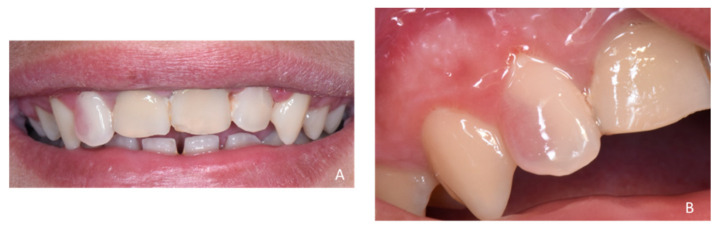
Intraoral views of the smile (**A**) and zoom on the pink spot lesion of tooth 12 (**B**).

**Figure 4:**
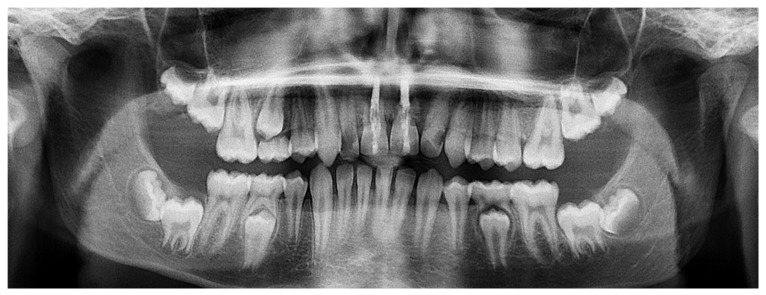
Orthopantomogram of the patient’s dental condition in April 2018.

**Figure 5:**
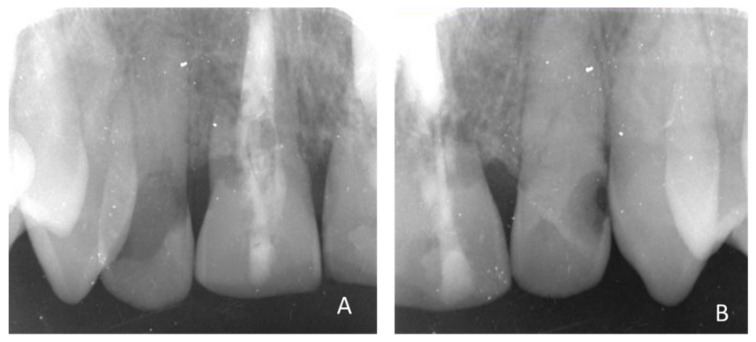
X-ray visualization of the invasive cervical resorptions of tooth 12 (**A**) and 22 (**B**).

**Figure 6:**
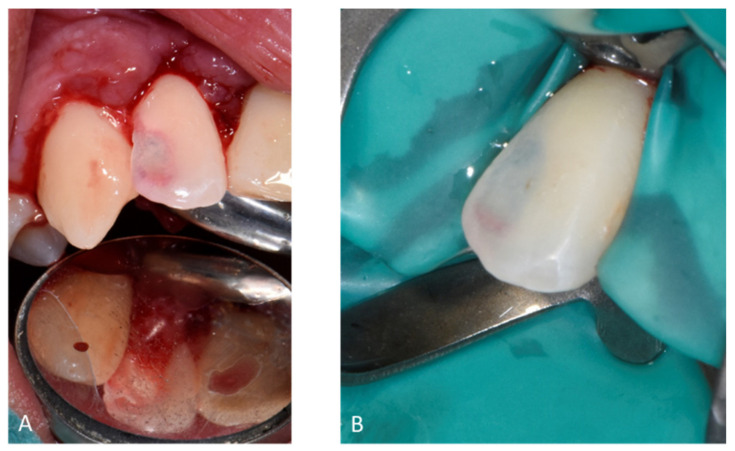
Per-operative endo-buccal views of 12. (**A**) After curettage and (**B**) after dam placement.

**Figure 7:**
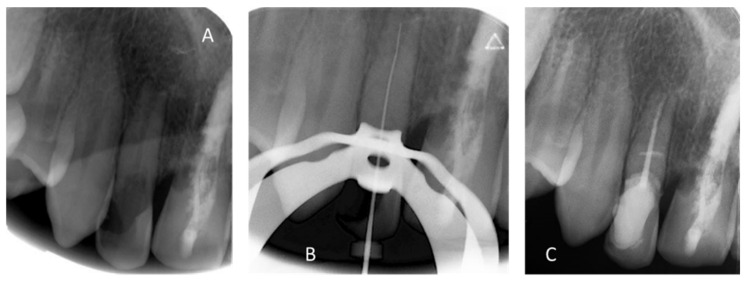
Pre (**A**), per (**B**) and post (**C**) retro-alveolar X-rays of the endodontic treatment of 12 (BioRoot™ and calibrated cone).

**Figure 8:**
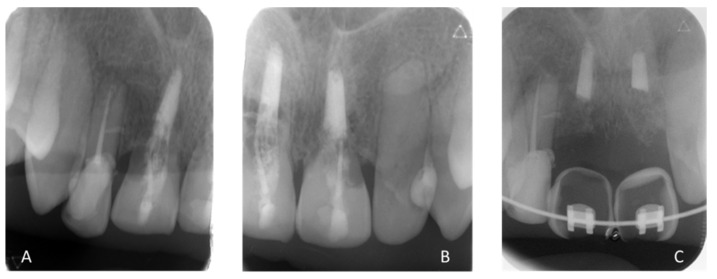
X-ray illustrating the state of inflammatory root resorption of (**A**) 11 and (**B**) 21, leading to their avulsion. (**C**) Remains of root canal filling material (gutta percha) in the bone after the avulsion of 11 and 21.

In August 2015, following a maxilla-facial trauma, a 10-year-old boy patient was admitted at the maxilla-facial department. The patient had no general health problems, was not taking any treatment, had no allergies and was not subjected to passive smoking. The young patient had a significant dental Class II malocclusion (Angle classification) due to skeletal Class II mandibular etiology (Ballard classification). Clinical and radiological examination revealed a complete extrusive luxation of 11 and 21 and intrusive luxation of 12 and 22. Practitioners decided to place a Dautrey arch. Two weeks later, the patient came to the dental emergency due to injuries caused by the Dautrey arch (Figure 1). Three months later, after removing Dautrey arch, a multi-attach treatment combined with Powerscop was put in place (Figure 2). Following the beginning of his orthodontic treatment, in December 2015, the patient’s attending dentist performed the endodontic treatment of 11 and 21, after the occurrence of root resorption consecutive to the absence of endodontic treatment immediately after the trauma. The patient was then referred to the endodontic department 3 years after trauma. The first treatment phase is described in Table 1 and Figure 3, Figure 4, Figure 5, Figure 6, Figure 7 and Figure 8.

### 2.2. Second Step: Surgical Phase

**Figure 9:**
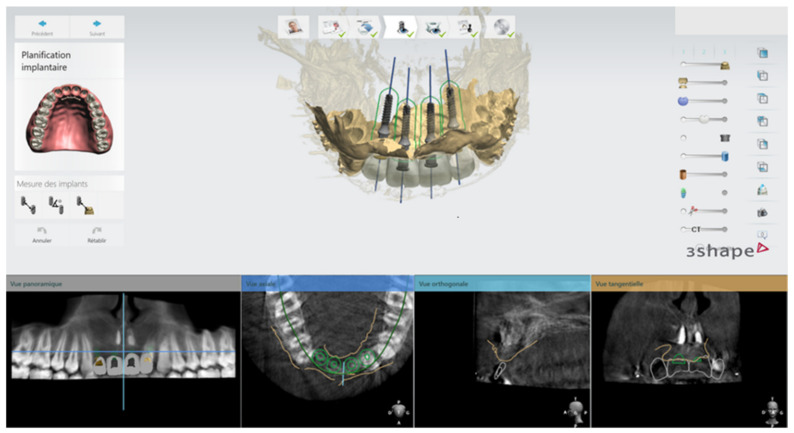
Screenshot of the implant-planning phase.

**Figure 10:**
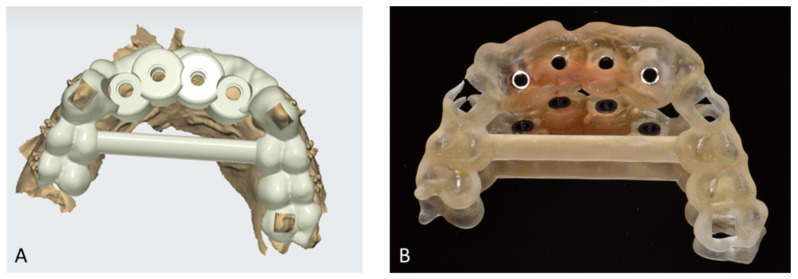
Screenshot of the surgical guide modeling (**A**) and printed surgical guide (**B**).

**Figure 11:**
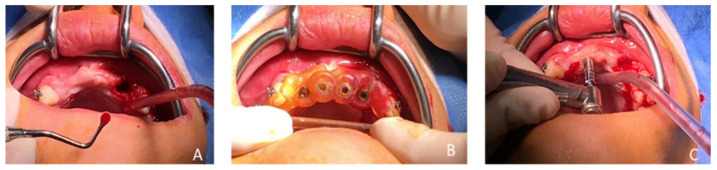
(**A**) Avulsion of tooth 22 and curettage of the alveolus, (**B**) trial of the surgical guide and (**C**) passage of the circular scalpel forest.

**Figure 12:**
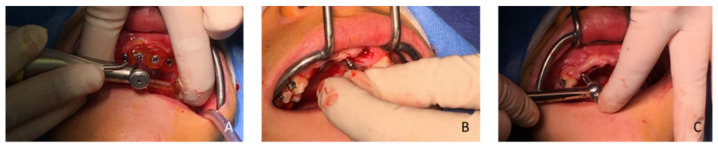
(**A**) Drilling (2.7 mm drill) of the central site (Flapless method), (**B**) placement of a parallelism gauge and (**C**) implant placement in tooth 12.

**Figure 13:**
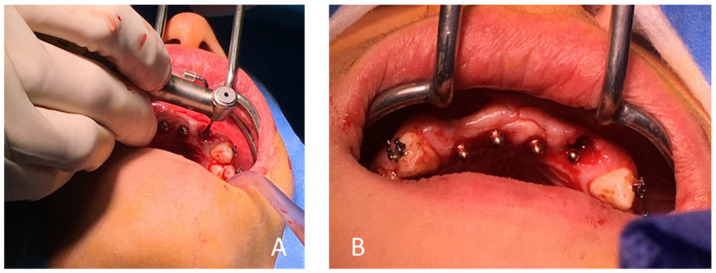
(**A**) Post-extraction drilling for the implant in tooth 22 and (**B**) the four installed OBI^®^ mini implants. Note that the position initially planned with the surgical guide and during the pre-implant planning of the implant in 22 had to be modified following the avulsion of the tooth, for better implant stability. Therefore, the drilling was not carried out with the guide, but hand free.

**Figure 14:**
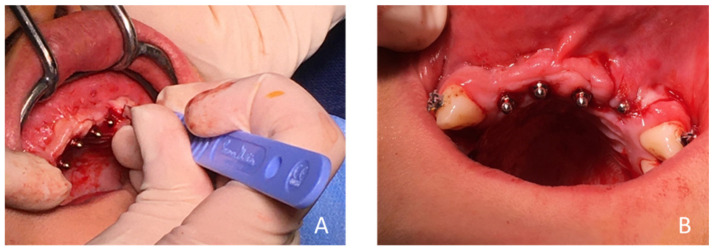
(**A**) Fabrication of a flap at tooth 22 to obtain a post-extraction edge-to-edge closure of the post-extraction site and (**B**) immediate post-operative endo-buccal view of the 4 mini OBI^®^ implants (4.0 resorbable sutures).

**Figure 15:**
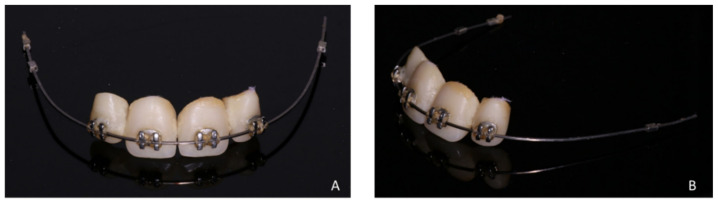
Front (**A**) and side (**B**) views of the orthodontic arch with the 4 prosthetic teeth replacing the maxillary incisal block.

In February 2020, following the avulsion of the tooth 12, clinical and radiographic examination revealed a bone deficiency in the buccal area. The cephalometric analysis from October 2019 showed that the patient was in his pubertal growth spurt [17]. Combined with the patient’s young age and his incomplete growth, this contraindicated the placement of conventional diameter implants. Another option for managing this clinical case was to maintain the gap between 13 and 23 by placing a removable prosthesis. However, there is some risk of blocking the jaw’s growth, as well as a lack of acceptance of the device. The patient chose not to have a removable prosthesis. The patient wanted a fixed, comfortable and esthetic solution. Bone capital had to be preserved for implant placement once growth was complete. It was therefore decided in agreement with the patient, his parents and the surgical and prosthetic teams, to use mini-implants. Four OBI^®^ (Eurotechnika, Sallanches, France) of a diameter of 2.7 mm type mini-implants were placed after avulsion of tooth 22. The OBI^®^ implants length ranged from 9 to 15 mm. An optical and a Cone Beam Computed Tomography (CBCT) impression were made to plan the surgery and print a surgical guide from the 3Shape Implant Studio software (Straumann, Basel, Switzerland) (Figure 9 and Figure 10). The implantation planning allowed to optimize the drilling axis of the mini implants according to the residual bone volume. After implant placement (Figure 11, Figure 12, Figure 13 and Figure 14), an orthodontic arch with four incisors was set up as a temporary solution (Figure 15).

### 2.3. Third Step: Prosthetic Phase

**Figure 16:**
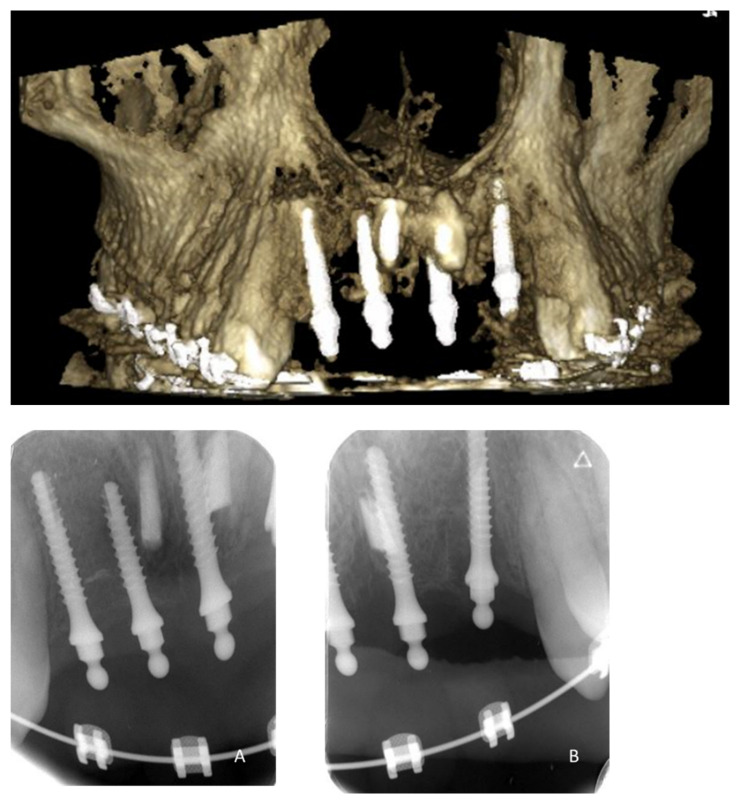
CBCT and retro-alveolar X-rays (**A**,**B**) of the 4 implants in place before the prosthetic phase.

**Figure 17:**
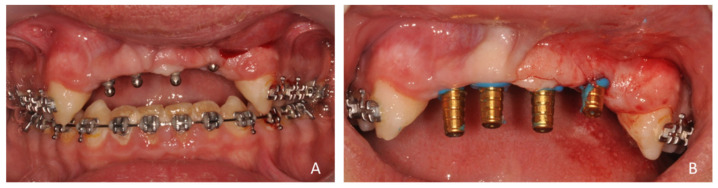
Endo-buccal views. (**A**) Implants in September 2020 and (**B**) light silicone stabilized copings on OBI^®^ ball implants.

After checking the bone osteo-integration of the implants (Figure 16), an Impregum™ (3M, Saint-Paul, MN, USA) impression was made for the maxilla and an alginate impression for the mandible (Figure 17).

**Figure 18:**
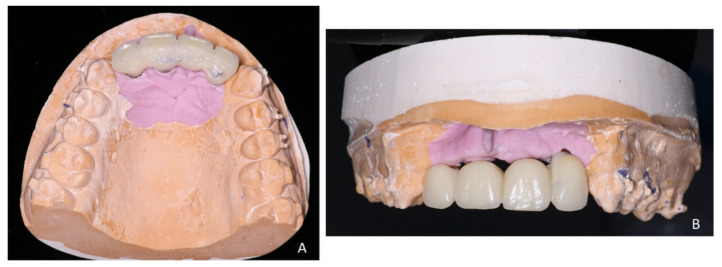
Bridge on model. (**A**) Palatal view and (**B**) vestibular view.

**Figure 19:**
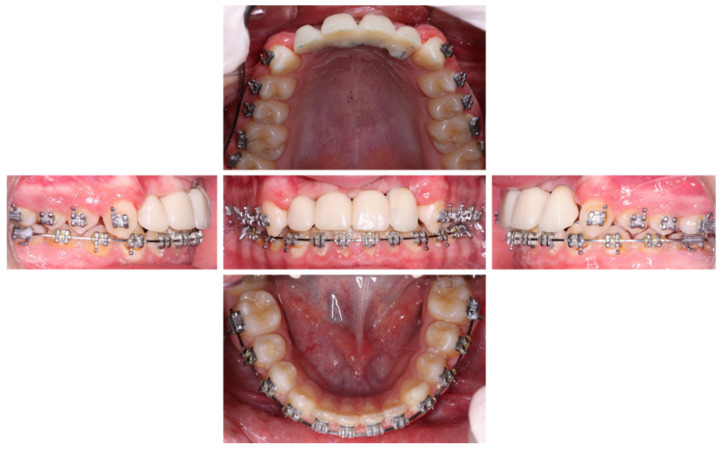
Inter- and intra-arch endo-buccal views, in profile and in occlusion, on the day of placement of the bridge.

**Figure 20:**
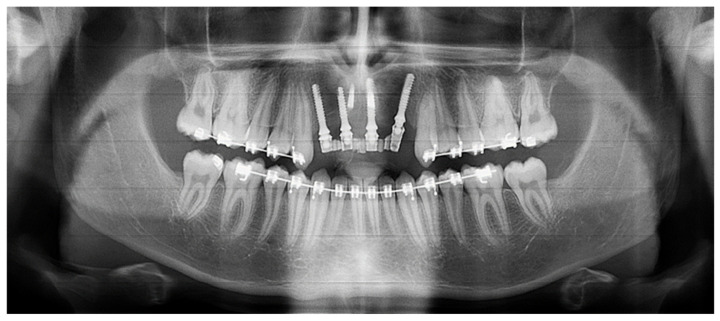
Panoramic view of the patient in November 2020, 5 months after implant placement.

**Figure 21:**
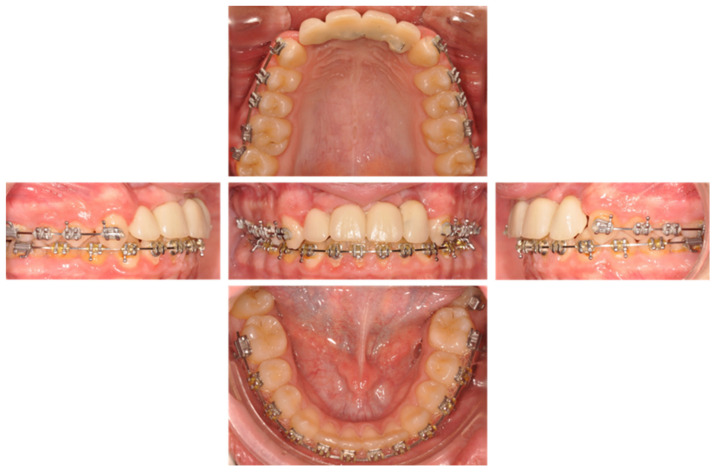
Inter- and intra-arch endo-buccal views, in profile and in occlusion, 2 months after placement of the anterior bridge.

**Figure 22:**
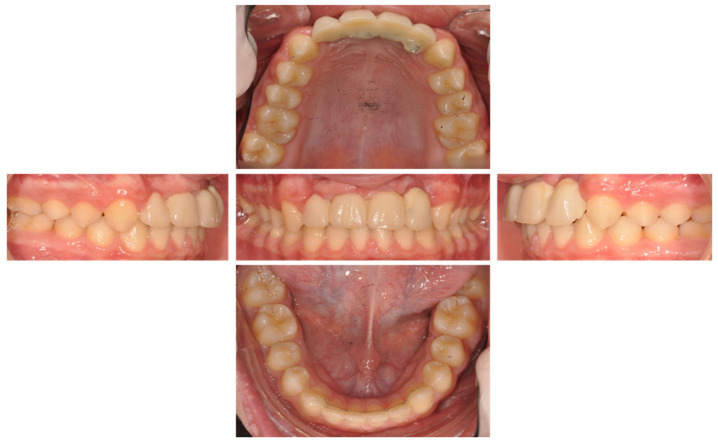
Removal of orthodontic arc: inter- and intra-arch endo-buccal views, in profile and in occlusion, 6 months after placement of the anterior bridge.

Then, a resin bridge (Figure 18) was sealed with glass ionomer cement (Fuji One GC company, Tokyo, Japan) (Figure 19). A control visit was realized at 2 (Figure 20 and Figure 21) and 6 months later (Figure 22).

## 3. Discussion

This clinical case aimed at describing the management of a dental trauma in an adolescent and the interest of using delaying procedures to allow the patient to complete growth. When the patient was referred to endodontic department, tooth 12 presented an important loss of coronal and radicular tissues due to invasive cervical resorption. The young age of the patient, the important destruction and the weak prognosis of the tooth would not allow preserving the tooth until adulthood. If academic endodontic treatment aims at preserving an asymptomatic tooth functional in the arch, the objective of “palliative” endodontic treatment could be preserving an asymptomatic nonfunctional tooth. “Palliative” endodontic treatment is achieved in special conditions. The “special conditions” reported in the article therefore include anatomical considerations, physiological considerations such as waiting for the end of growth in a young patient or healing of an apical bone lesion allowing an implant to be placed under better conditions, or financial conditions. The goal of endodontic treatment in this situation was to keep the tooth as long as possible for placing implants in good conditions. The first delaying procedure was the palliative endodontic treatment of tooth 12.

Periodic follow-up was implemented but did not forestall complications. Both lateral incisors developed a “pink spot”, leading to an unfavorable prognosis. The tooth 12 was filled with a biocompatible and bioactive cement (BioRoot™ (Septodont, Saint-Maur-des-Fossés, France) mineralized tissue inducer), watertight and with antimicrobial properties [18]. Thermocompacting the Gutta was therefore not necessary, which was interesting in the case of short and fragile roots. The occurrence of suppurated chronic apical periodontitis on tooth 12 led to endodontic retreatment. As the root canal retreatment failed, no apical resection surgery was carried out to prevent bone loss to allow for future implant rehabilitation. Likewise, after the extractions of 11 and 21, root canal material (gutta percha) was left in the socket to avoid further alveolar destruction from curettage and alveolectomy. The objective was to preserve bone volume while waiting for the alveolar graft. Another reason was that the biocompatibility of the gutta percha in the bone structure did not require immediate removal [19,20]. The gutta percha is set to be removed at the time of bone graft.

The failure of the endodontic and restorative treatments indicated avulsions of tooth 12 and tooth 22 for prosthetic reason. The question of how to replace the missing tooth in a patient in his pubertal growth spurt was raised.

A conventional implant could be placed in patients in growth stage in the case of absence of one or more teeth because of congenital disorder, in the case of alveolar cleft, and in the case of trauma [12,13,21]. In this case, the available bone volume is low because of vertical and horizontal resorption of bone: bone graft and periodontal arrangements will be necessary. There is no age recommendations for bone graft, or for the number and the position of implant required [13]. There is also a risk of infra-occlusion of conventional implant due to the vertical growth of anterior maxilla that is influenced, up to 18 years old, by vertical growth [12,13]. For these reasons, conventional diameter implants were contraindicated and the placement of the OBI^®^ implant was chosen as a second delaying procedure. Mini-implants should be an alternative therapeutic solution to conventional implants allowing bone stimulation and prevention of resorption to await the end of the growth. Mini-implants (diameter from 1.8 mm to 3 mm) are often used in the case of limited bone anatomy and suitable for one-stage surgical placement under guided surgery on healed areas. Under these conditions, they have good primary stability. Their one-piece design avoids a gap between the implant and the prosthetic abutment, with favorable consequences for mucosal healing. The survival rate of the mini implant is high [22,23] and received approval for long-term usage for complete dentures, removable partial dentures, and multi-unit fixed prosthetics [24]. Fixed supra-mini-implant prosthesis allowed the rehabilitation of esthetics and function.

Once growth is completed, the next step in oral rehabilitation will be one or more bone grafts and periodontal arrangements. Two conventional implants will be necessary to replace the four mini-implants.

## 4. Conclusions

The management of early treatment failure of trauma in adolescents may be pluridisciplinary and take into consideration the endodontic approach, the available bone volume and the pubertal growth stage. As presented in this clinical case, the combination of delaying procedures such as “palliative” endodontic treatment and mini-implants allowed the management of trauma in growing children.

## Authors Contributions

P.-Y.C.: treatment planning; N.D. and P.-Y.C.: supervision of restorative and endodontic treatments; L.D.: supervision of surgical phase; E.N. and J.-L.V.: supervision of prosthetic phase; M.-L.M.-S.: realization of the steps of the treatment; M.-L.M.-S., P.-Y.C. and E.N.: writing—original draft preparation; M.-L.M.-S., P.-Y.C. and E.N.: writing—review and editing. All authors have read and agreed to the published version of the manuscript.

## Figures and Tables

**Table 1:** Initial step of management of dental treatment.

Treatment				
Date/Events	Teeth			
	12 (Lateral Upper Right Incisor)	11 (Central Upper Right Incisor)	21 (Central Upper Left Incisor)	22 (Lateral Upper Left Incisor)
August 2015: Maxilla-facial trauma, Dental traumatology, Alveolar fracture	Intrusive luxation	Complete extrusive luxation		Intrusive luxation
August 2015: General Anesthesia	Reducing of extrusive luxations			
	Dautrey arch placed with inter-dental fixation using 2.0 steel wire between both first upper molar teeth to allow reduction in the associated alveolar fracture			
October 2015: Orthodontics consultation	Severe mobility and root resorption			
	Removal of the Dautrey arch and replacement with a fixed multi-fastener maxillary appliance			
December 2015 (Dentist treatment)	Endodontic treatment with calcium hydroxide and Gutta Percha			
April 2018	“pink spot” lesions/invasive cervical resorption of class III according to Heithersay classification [15,16]/negative response to vitality test	Slight mobility/complete root inflammatory resorption around the gutta percha filling		“pink spot” lesions/invasive cervical resorption of class II according to Heithersay classification [15,16]/negative response to vitality test
	Figure 3, Figure 4 and Figure 5			
May 2018	Buccal and palatal flap of upper right anterior teeth without discharge/curettage of the granulation tissue/endodontic (lesion was of interest to the pulp)/restoration with a composite resin under dam/endodontics treatment with sodium hypochlorite irrigation and obturation at BioRoot™ and calibrated cone			Buccal flap without discharge from upper left anterior teeth/curettage of the granulation tissue/restoration with a composite resin under dam
	Preservation of the bone capital until the implant solution			
	Figure 6 and Figure 7			
May 2019: Orthodontic treatment for skeletal class II	Avulsion			
	Maintaining space for future implant replacement with resin crown temporization on the orthodontic arch			
	Figure 8			
Summer 2019	Two infectious episodes despite an endodontic re-treatment			
January 2020	Avulsion			

## Data Availability

Non-applicable.
